# A Sarcopenia Index Derived from Malnutrition Parameters in Elderly Haemodialysis Patients

**DOI:** 10.3390/nu15051115

**Published:** 2023-02-23

**Authors:** M. L. Sánchez-Tocino, S. Mas-Fontao, C. Gracia-Iguacel, M. Pereira, I. González-Ibarguren, A. Ortiz, M. D. Arenas, E. González Parra

**Affiliations:** 1 Fundación Renal Íñigo Álvarez de Toledo, 28003 Madrid, Spain; 2 Servicio de Nefrología e Hipertensión, Fundación Jiménez Díaz, 28040 Madrid, Spain; 3 Spanish Biomedical Research Centre in Diabetes and Associated Metabolic Disorders (CIBERDEM), 28029 Madrid, Spain; 4 Servicio de Geriatría, Hospital Universitario de Guadalajara, 19002 Guadalajara, Spain

**Keywords:** hemodialysis, sarcopenia, malnutrition, aging

## Abstract

(1) Background: Persons with chronic kidney disease may have sarcopenia characterized by the loss of muscle mass and loss of muscle strength. However, EWGSOP2 criteria to diagnose sarcopenia are technically challenging, especially in elderly persons on hemodialysis. Sarcopenia may be associated with malnutrition. We aimed at defining a sarcopenia index derived from malnutrition parameters for use in elderly haemodialysis patients. (2) Methods: A retrospective study of 60 patients aged 75 to 95 years treated with chronic hemodialysis was conducted. Anthropometric and analytical variables, EWGSOP2 sarcopenia criteria and other nutrition-related variables were collected. Binomial logistic regressions were used to define the combination of anthropometric and nutritional parameters that best predict moderate or severe sarcopenia according to EWGSOP2, and performance for moderate and severe sarcopenia was assessed by the area under the curve (AUC) of receiver operating characteristic (ROC) curves. (3) Results: The combination of loss of strength, loss of muscle mass and low physical performance correlated with malnutrition. We developed regression-equation-related nutrition criteria that predicted moderate sarcopenia (elderly hemodialysis sarcopenia index-moderate, EHSI-M) and severe sarcopenia (EHSI-S) diagnosed according to EWGSOP2 with an AUC of 0.80 and 0.866, respectively. (4) Conclusions: There is a close relationship between nutrition and sarcopenia. The EHSI may identify EWGSOP2-diagnosed sarcopenia from easily accessible anthropometric and nutritional parameters.

## 1. Introduction

The word sarcopenia derives from Greek, meaning a scarcity (penia) of flesh (sarx). Irwin Rosenberg first used the term sarcopenia in 1988, identifying a clinical condition characterized by the loss of skeletal muscle mass in the context of ageing [1]. However, the definition of sarcopenia has evolved over the years, and several definitions have been proposed. The available definitions always include muscle mass; in addition, some include muscle strength and most include physical performance [2,3,4,5,6]. Sarcopenia is primarily associated with old age [7], and in 2016, it was listed as a disease in the International Classification of Diseases (CIE-10, MC version) with the code M62.84 [8,9].

In February 2018, the European Working Group on Sarcopenia revised and updated its definition of sarcopenia (EWGSOP2), which is the most widely used, in a new consensus document [10]. Sarcopenia is defined by EWGSOP2 as a skeletal muscle disease understood as the loss of muscle mass and loss of strength. Therefore, sarcopenia combines the concepts of myopenia (decreased muscle mass) and dynapenia (decreased muscle strength). Assessment of sarcopenia by EWGSOP2 may be technically challenging, especially for haemodialysis (HD) patients, requiring techniques such as dual-energy X-ray absorptiometry (DXA), bioelectrical impedance analysis (BIA), magnetic resonance imaging (MRI) or computed tomography (CT), which are not frequently available in routine clinical settings for use for this purpose, in addition to functional evaluations that may not be possible for some HD patients. There is thus a need for simpler parameters that guide clinicians in peripheral or low-resource HD units.

The prevalence of sarcopenia in HD patients ranges from 4% to 64% [11,12,13] depending on the diagnostic criteria applied. Following the EWGSOP2 definition, we previously reported a prevalence of sarcopenia in elderly HD patients of 20% [14].

In chronic kidney disease (CKD) patients, multiple causes lead to an imbalance between muscle synthesis and catabolism, and the term uraemic myopenia has been coined [15,16,17,18,19,20,21,22,23,24,25]. This leads to decreased muscle quantity, an altered muscle structure, muscle atrophy and reduced muscle strength [26,27]. However, there is not always a linear relationship between skeletal muscle size and strength. These patients may have a disproportionate loss of strength despite having muscle mass within normal limits [14].

Chronic renal failure is characterized by nutritional disturbances and systemic inflammation accompanied by increased catabolism, which increase morbidity and mortality. Malnutrition and inflammation may contribute to sarcopenia. However, there are several definitions of malnutrition in CKD patients that include muscle assessment, and, thus, sarcopenia and malnutrition are interconnected concepts [28]. The International Society of Renal Metabolism and Nutrition has defined protein energy wasting as a pathological state where there is a continued depletion of both protein stores and energy reserves [29,30], including the simultaneous loss of muscle. In our population, 37% of HD patients had protein energy wasting [31]. The management of sarcopenia includes optimizing the diet [32].

In the present study, we explored the relationship between biochemical and body composition criteria for the diagnosis of malnutrition and developed, from an HD population over 75 years of age, a simple index, the elderly hemodialysis sarcopenia index (EHSI), that provides information on sarcopenia diagnosed according to EWGSOP2 criteria with a high area under the curve (AUC).

## 2. Materials and Methods

### 2.1. Study Design

A retrospective study was conducted on patients in chronic hemodialysis in three outpatient units and one hospital of the Fundación Renal Íñigo Álvarez de Toledo in Spain (Hospital Fundación Jiménez Díaz de Madrid and outpatient units Centro Santa Engracia (Madrid), Centro de Bejar and Centro de Ciudad Rodrigo (Salamanca)) in February 2019, i.e., prior to the coronavirus disease 2019 (COVID-19) pandemic. Inclusion criteria were age from 75 to 95 years, the capability to perform physical fitness assessment tests or dynamometry and patients who had been on HD for more than 3 months. All patients were dialyzed for 210 min per session, 3 days a week, with a maximum blood flow of 300 mL/min with conventional dialysis.

The study was approved by the ethics committee of the Hospital Universitario Fundación Jiménez Díaz (acta no. 03/19) and complied with the standards recognized by the Declaration of Helsinki of the World Medical Association, as well as the Standards of Good Clinical Practice, in addition to compliance with Spanish legislation on biomedical research (Law 14/2007). All participants signed informed consent for their participation.

### 2.2. Study Variables

Both sarcopenia and nutritional status were assessed. 

The EWGSOP2 three-stage diagnostic work-up was used to assess sarcopenia:

(A) Probability: Loss of grip strength was determined by the hand grip (HG) using an electric CAMRY^®^ Model EH101 dynamometer with the participant standing with their arm extended along the body and not supporting it or moving the wrist. Maximum grip strength was maintained for 3 s, with a rest of 1 min between each repetition, making two attempts in both arms. The strongest grip achieved by the dominant arm was the one used for the study [33]. The cutoff points that determine the probability of sarcopenia (dynapenia) are strength less than 27 kg in men and less than 16kg in women [34].

(B) Confirmation: An assessment was performed of the appendicular skeletal muscle mass by bioimpedance (ASM) defined as the sum of the muscle mass of the four limbs [7]. A MALTRON^®^ bioimpedance device, model BioScan touch i8, was used in the second HD session of the week, in the second hour of treatment, since the device allows an assessment while HD is underway. The Maltron bioscan 916 device is validated for assessing body composition in situations when extracellular water (ECW) is changing. Thanks to this software, dry weight can be calculated when no more volume is extracted from the ECW despite ongoing ultrafiltration, and no change in resistance is observed [35]. The cutoff points to diagnose sarcopenia as the loss of muscle mass (myopenia) are ASM less than 20 kg in males and less than 15 kg in females [36].

(C) Severity: Physical performance was assessed. It was determined by the variable gait speed (GS) evaluated as the time required to walk 4 m and expressed in meters per second, considering whether any assistance (cane, walker, another person, etc.) was required to maintain balance while walking. Walking included one meter in front and one meter behind the four meters that were assessed so that the results would not be influenced by acceleration and deceleration [37]. The cutoff point that determines severe sarcopenia is a speed <0.8 m/s [6].

#### Nutritional Status Was Assessed by the Malnutrition–Inflammation Score (MIS), Anthropometric Variables, Biochemical Variables and Body Composition

(A) The MIS is a fully quantitative score adapted from the subjective global assessment used for the early identification of malnutrition–inflammation. The MIS is associated with nutritional parameters, inflammatory status and mortality [38]. It is a validated questionnaire for the dialysis population consisting of 10 components, each scored from 0 to 3, for a range of values from 0 to 30: weight change, appetite, gastrointestinal symptoms, functional capacity related to nutritional factors, comorbidities including years on dialysis, subcutaneous fat loss, muscle mass, body mass index (BMI), serum albumin and total iron binding capacity. Above 10 points, patients have extreme malnutrition; from 7 to 10 points, very severe malnutrition; from 5 to 7 points, moderate–severe malnutrition; from 2 to 5 points, mild–moderate malnutrition; and below 2 points, normonutrition [39].

(B) Anthropometric variables. The BMI was determined as weight (kg)/height (m)^2^. BMI is used as a marker of obesity. In Caucasian populations, the BMI cutoff point for obesity is 30 kg/m^2^ [40]. A weight loss of at least 5% in 12 months or less or a BMI < 20 kg/m^2^ is diagnostic of cachexia [41].

Brachial circumference (BC) is an indicator of decreased tissue protein reserves used in older adults, as it is easy to measure. Its use in conjunction with other measurements, such as the tricipital and bicipital folds, may provide more complete information on caloric-protein reserves [42].

The waist-to-hip ratio (WHI) was assessed from waist and hip circumferences. The WHI assesses intra-abdominal fat. The waist circumference (WC) and WHI better assess cardiovascular risk than the BMI [43].

Tricipital, abdominal and subscapular skin folds indicate total body fat. They were assessed with a caliper by calculating the average value of 3 measurements in millimeters. In the elderly population, skin folds may be less reliable [44].

(C) Analytical variables: The following parameters were measured: serum albumin, proteins, hemoglobin, hematocrit, cholesterol, lymphocytes, protein catabolism rate and 25OH vitamin D [41,45,46]. In addition, dialytic efficacy was assessed using Daurgidas’ Kt/Vurea [47].

(D) Body composition was determined by bioimpedance, assessing muscle mass, fat mass, fat-free mass, extracellular mass, total cell mass, body water, extracellular water, intracellular water, overhydration and hydration of lean mass. Body impedance (Z) is a function of 2 components or vectors—resistance (R) and reactance (Xc)—according to the equation Z2 = R2 + Xc2. R represents the resistance of the tissues to the passage of an electric current and Xc is the additional opposition due to the capacitance of these tissues and cell membranes [48]. Electrical conductivity is higher in lean tissue than in adipose tissue, since the former contains almost all the water and electrolytes [49].

### 2.3. Statistics

Statistical analyses were performed with the IBM SPSS Statistics v20 program (IBM, Armonk, NY, USA). Quantitative variables are presented as the mean and standard deviation or median (interquartile range). Qualitative variables are presented as absolute numbers and percentages. Student’s *t*-test or Wilcoxon test was used to compare two quantitative variables, and Pearson’s or Spearman coefficient was used for correlation studies. The association between qualitative variables was evaluated using the chi-square test. The level of statistical significance was established at *p* ≤ 0.05. Binomial logistic regressions were used to define the combination of anthropometric and nutritional parameters that best predict moderate or severe sarcopenia according to EWGSOP2, and performance for moderate and severe sarcopenia was assessed by the area under the curve (AUC) of receiver operating characteristic (ROC) curves.

## 3. Results

Table 1 shows the characteristics of the study population, also divided into men and women. Men and women differed in Kt/Vurea and anthropometry, as expected. There were also differences in lean and fluid composition but not in sarcopenia and/or frailty criteria.

Table 2 describes the analytical, clinical, body composition and nutrition data of participants in two groups with normonutrition/mild–moderate malnutrition or severe/extreme malnutrition according to the MIS (≤5 and >5 points, respectively). Malnourished patients had a higher KTVurea, as expected due to the lower muscle mass, urea distribution volume, albumin and weight, less muscle and body water and greater frailty measured with FRAIL.

Table 3 shows the correlations between nutritional parameters, including the MIS, with the sarcopenia criteria used by EWGSOP2 for probability, confirmation and severity. There were no significant correlations of nutritional parameters with those of the probability and confirmation of sarcopenia measured by muscle mass. However, severe sarcopenia correlated with parameters of malnutrition. The combination of loss of strength, muscle mass and physical performance correlated with malnutrition.

### Prediction of Sarcopenia

In subjects presenting sarcopenia criteria according to EWGSOP2, a mild correlation with nutritional parameters was observed; however, the presence of malnutrition was not significantly associated with the presence of sarcopenia. Moderate sarcopenia was observed in 30% of normonourished patients and in 50% of malnourished patients. Severe sarcopenia was observed in 20% of the normonourished population and in 40% of those who are malnourished.

We used binomial logistic regressions to define the combination of anthropometric and nutritional parameters that best predicts moderate or severe sarcopenia according to EWGSOP2.

A combination of gender, age, serum albumin, phosphate and cholesterol (3.0055 + 1.2218[gender] − 0.1358[age] + 0.5977[albumin] + 0.7246[phosphate] + 0.0202[cholesterol]) predicted moderate sarcopenia with a specificity of 87% and sensitivity of 61%, yielding an AUC of 0.8 with a precision of 0.7119 for a cutoff value of 0.745. We called this the elderly hemodialysis sarcopenia index-moderate, EHSI-M.

Severe sarcopenia was predicted with a specificity of 94% and a sensitivity of 76% (AUC 0.866 and a precision of 0.815 for a cutoff of 0.71) by 12.3261 − 1.8643[gender] − 0.1830[age] − 0.0430[MIS] + 0.257[cholesterol] + 1.1139[lymphocytes] + 1.1987[subscapular fold]. We called this the elderly hemodialysis sarcopenia index-severe, EHSI-S.

## 4. Discussion

The present study confirmed the close relationship between nutrition and sarcopenia in patients over 75 years of age on hemodialysis. Additionally, we provide a tool, the elderly hemodialysis sarcopenia index (EHSI), that may be useful to estimate the risk of sarcopenia according to EWGSOP2 in elderly hemodialysis patients. This tool will be useful for centers that do not have regular access to bioimpedance that can be performed during the hemodialysis session for monitoring sarcopenia. A holistic intervention, including a nutritional intervention, is important to avoid sarcopenia and the effects of sarcopenia on frailty, quality of life, dependence and mortality in these patients [50].

Nutritional parameters did not correlate with the suspicion and confirmation of sarcopenia according to EWGSOP2 as assessed by the loss of strength and muscle mass. However, severe sarcopenia (loss of strength, mass and gait speed) correlated with different parameters of malnutrition. Only the combination of loss of strength, muscle mass and physical performance correlated with malnutrition. These data are consistent with prior data suggesting that malnutrition contributed to sarcopenia/severe sarcopenia in 315 Asian maintenance HD patients by reducing muscle mass, strength and physical performance [51]. We observed moderate sarcopenia (loss of strength and loss of muscle mass) in 30% of normonourished patients and in 50% of malnourished patients. Severe sarcopenia was found in 20% of normonourished patients and in 40% of those malnourished.

The origin of sarcopenia is multifactorial. Uremic patients may lose muscle function independent of adequate muscle mass [52]. Muscle mass and strength are associated with exercise [53] but not with adequate nutrition without exercise.

Acidosis increases the risk of sarcopenia in CKD patients [54]. Alkali supplementation to treat acidosis increased lean body mass[54], mid-arm muscle circumference and lower limb muscle strength [55]. Diets rich in vegetables, fruits and greens also improved acidosis [56,57].

A high protein intake (1 to 1.2 g/kg/day) improved muscle health and prevented sarcopenia in elderly people with normal renal function [58,59]. However, this relationship has not been demonstrated in CKD patients, who may be prescribed protein intake restriction (0.55–0.6 g/kg/day, or 0.6–0.8 g/kg/day in diabetics as per 2020 guidelines) to slow CKD progression, which may negatively impact muscle mass and function [60]. In CKD patients on dialysis, a higher intake (1.0–1.2 mg/kg) is recommended [16].

Despite this reflection, the latest revision published in 2020 of the KDOQI clinical practice guidelines for nutrition in patients with CKD [60] maintains the recommendation of a protein intake in predialysis stages of 0.55–0.6 g/kg/day. We believe that in elderly patients with stage 3–5 CKD, the risk of malnutrition and sarcopenia should be assessed as a differentiating value for recommending an intense restriction of protein intake, independent of the progression, or lack thereof, of the deterioration of renal disease. 

A systematic review and network meta-analysis revealed the benefits of exercise on muscle strength. However, the combination of exercise and nutrition did not improve muscle strength and physical performance above exercise alone [61]. In elderly HD patients, EWGSOP2 sarcopenia criteria improved with muscular exercise [62].

Given that diagnosing sarcopenia using EWGSOP2 criteria may be time consuming and technically challenging, efforts have been made to identify sarcopenia using simpler analytical and clinical criteria. Recently, a sarcopenia index was described in persons with cardiovascular disease. The sarcopenia index includes five independent factors (sex, age, BMI, adiponectin and sialic acid) and had a high accuracy in ROC curve analysis (sensitivity of 94.9% and specificity of 69.9%) [63]. However, it still contained uncommon analytes (adiponectin and sialic acid) that may render the index of little value in patients with kidney failure, as their circulating levels increase as the glomerular filtration rate decreases [1,2]. In this equation, there is a negative relationship between age and the likelihood of sarcopenia; older patients possibly have less sarcopenia, as a protective factor, so those with more sarcopenia may be less likely to reach older ages. We have now developed two regression formulas to test for sarcopenia in elderly patients on hemodialysis using easily accessible clinical data. EHSI-M and EHSI-S may be useful for easy patient monitoring for sarcopenia in low-resource centers, which represent the majority of global HD units, allowing early intervention. Additionally, even high-resource centers may not have as regular access as needed to tools such as dual-energy X-ray absorptiometry. Resonance imaging and computed tomography may be needed to diagnose sarcopenia according to EWGSOP2 criteria, while bioimpedance may be problematic in HD patients given the rapid fluid changes during dialysis and the desire of patients to leave for home as early as possible after HD sessions.

Several limitations should be acknowledged, including the need for external validation of the equations to assess sarcopenia in persons from different ancestries and continents. For this purpose, we provide easy calculators that allow centers across the world to test the EHSI-M and EHSI-S and prospectively explore their prognostic value and their response to interventions. In addition, we used BIA instead of the gold standard dual-energy X-ray absorptiometry, resonance imaging and computed tomography to assess EWGSOP2. Among the strengths, tests were performed by highly trained personnel, the BIA techniques employed allowed assessments during the HD session and we provide data in an understudied but growing population, very elderly HD patients, for whom the performance of tests needed for EWGSOP2 may be problematic.

## 5. Conclusions

In conclusion, there was a close relationship between nutrition and sarcopenia. A tool is provided, developed from nutrition and simple analytical criteria, that may identify moderate and severe sarcopenia according to EWGSOP2 criteria and that can be used to monitor patients at a low cost in terms of time and technical resources. The progression of malnutrition and/or sarcopenia should be a factor to be considered when restricting protein intake in patients with ACKD G3-5.

## Figures and Tables

**Table 1:** Descriptive analysis of the main clinical and analytical variables. *p* values refer to the comparison between males and females. Mean ± SD.

Variables	All (n = 60)	Female (n = 19)	Male (n = 41)	*p*	Normal Range
Age	81.85 (5.58)	83.00 (5.23)	81.32 (5.72)	n.s	
Female, n (%)	19 (31.7)	19 (100.0)	41 (100.0)	n.s	
Dialysis vintage (months)	49.76 (40.4)	53.22 (42.8)	48.16 (39.7)	n.s	
Catheter (CVC), n (%)	15 (25.0)	5 (26.3)	10 (24.4)	n.s	
Diabetes, n (%)	20 (60.6)	3 (68.4)	23 (56.1)	n.s	
Malignancy, n (%)	39 (65.0)	15 (78.9)	24 (58.5)	n.s	
Kt/V urea	1.80 (0.38)	2.01 (0.30)	1.70 (0.37)	<0.01	>1.3 M; >1.4 F
Albumin (g/dL)	3.66 (0.48)	3.59 (0.60)	3.70 (0.41)	n.s	3.4–5.4
Total protein (g/dL)	6.10 (0.67)	5.99 (0.81)	6.14 (0.60)	n.s	6–8.3
Hemoglobin (g/dL)	11.36 (1.06)	11.26 (1.13)	11.40 (1.03)	n.s	M: 13.8–17.2; F: 13.1–15.1
Phosphate (mg/dL)	4.6 (1.1)	4.5 (1.2)	4.6 (1.0)	n.s	2.5–4.5
CRP (mg/L)	1.60 (2.78)	1.68 (2.65)	1.57 (2.87)	n.s	<5
25-OH vitamin D (ng/mL)	21.52 (13.1)	22.31 (13.82)	21.1 (12.95)	n.s	20–40
Total cholesterol (mg/dL)	137.7 (31.1)	144.95 (32)	134.3 (30.5)	n.s	<200
BMI (kg/m^2^)	25.20 (3.65)	24.06 (3.97)	25.73 (3.41)	0.05	18.5–24.5
Brachial perimeter (cm)	26.32 (2.82)	25.70 (3.11)	26.62 (2.66)	n.s	Male > 25.74; Female > 24.5
Waist perimeter (cm)	92.78 (10.41)	83.88 (10.08)	97.01 (7.58)	<0.01	Male < 95; Female < 82
Hip perimeter (cm)	100.58 (7.23)	98.39 (7.56)	101.61 (6.93)	n.s	Male < 100; Female < 80
Waist hip index	0.92 (0.08)	0.85 (0.06)	0.96 (0.07)	<0.01	Male < 1; Female < 0.8
Tricipital fold (cm)	1.19 (0.43)	1.41 (0.46)	1.09 (0.38)	0.01	Male < 1.5; Female < 1.9
Subscapular fold (cm)	15.40 (7.10)	13.22 (7.61)	16.43 (6.70)	0.05	Female 17.6 mm; Male 15.95 mm
Abdominal fold (cm)	18.39 (6.35)	15.77 (4.39)	19.80 (6.85)	0.05	Male 19.8 mm; Female 22.2
Muscle mass (kg)	19.27 (3.82)	15.57 (1.87)	20.99 (3.23)	<0.01	>20
Fat mass (kg)	22.63 (5.86)	22.01 (7.40)	22.92 (5.08)	n.s	
Fat mass (%)	34.04 (6.27)	38.10 (6.94)	32.15 (4.98)	<0.01	<30%
FFM (kg)	43.78 (8.59)	34.59 (4.01)	48.04 (6.55)	<0.01	
FFM %	65.96 (6.27)	61.90 (6.94)	67.84 (4.98)	<0.01	<70%
TBW (L)	32.42 (6.52)	25.70 (3.16)	35.53 (5.18)	<0.01	<55%
ECW (L)	15.55 (3.36)	12.46 (1.96)	16.98 (2.88)	<0.01	<45%
ICW (L)	16.85 (3.29)	13.24 (1.33)	18.52 (2.47)	<0.01	<55%
ECW/ICW	0.92 (0.08)	0.94 (0.09)	0.91 (0.08)	n.s	0.5–1
BCM (kg)	22.83 (4.43)	18.53 (2.26)	24.82 (3.71)	<0.01	<60
ECM (kg)	19.47 (3.87)	14.97 (1.68)	21.56 (2.59)	<0.01	<40
FFMH (%)	73.94 (1.88)	74.26 (2.04)	3.79 (1.81)	n.s	<75
Fluid excess (L)	1.06 (1.49)	0.76 (1.22)	1.20 (1.60)	n.s	
HG criteria, n (%)	15 (25.0)	6 (31.6)	9 (22.0)	n.s	Male > 27 kg; Female > 16 kg
ASM criteria, n (%)	36 (60.0)	13 (68.4)	23 (56.1)	n.s	Male > 20 kg; Female > 15 kg
GS criteria, n (%)	18 (30.0)	7 (36.8)	11 (26.8)	n.s	>0.8 m/s
MIS (pts)	6.02 (3.81)	6.00 (3.21)	6.02 (4.09)	n.s	<5

VA: vascular access, CVD: cardiovascular disease, FFM: fat-free mass, TBW: total body water, ECW: extracellular water, ICW: intracellular water, BCM: body cellular mass and ECM: extracellular mass. FFMH: fat-free mass hydration. HG: grip strength by dynamometry, ASM: appendicular skeletal muscle mass, GS: gait speed and MIS: malnutrition–inflammation score. n.s: no significant.

**Table 2:** Analytical, clinical, body composition and nutrition data of participants with normonutrition or malnutrition according to the MIS (malnourished if >5 points).

	Normonutrition (n = 32)	Malnutrition (n = 28)	*p*
Age (years)	81.0 (76.4–86.0)	81.0 (78.0–87.0)	n.s
Female, n (%)	25% 8/32	40% 11/28	n.s
Dialysis vintage (months)	34.3 (12.2–59.1)	52.1 (26.6–85.3)	n.s
Cause of CKD, n (%)			n.s
Diabetes	22% 7/32	39%% 10/28	
Vascular	22% 7/32	18% 5/28	
Glomerular	6% 2/32	3% 1/28	
Interstitial	3% 1/32	11% 3/28	
Undetermined	41% 13/32	21% 6/28	
Others	6% 2/32	11% 3/28	
Catheter, n (%)	28% 9/32	21% 6/28	n.s
Diabetes, n (%)	63% 20/32	57% 16/28	n.s
Cardiovascular disease, n (%)	34% 11/32	32% 9/28	n.s
Malignancy, n (%)	75% 24/32	54% 15/28	n.s
Kt/Vurea	1.6 (1.4–1.9)	2.0 (1.8–2.1)	*p* < 0.01
Albumin (g/dL)	3.8 (3.7–4.0)	3.6 (3.3–3.9)	*p* < 0.01
Total protein (g/dL)	6.4 (6.1–6.5)	6.0 (5.5–6.4)	n.s
Hemoglobin (g/dL)	11.4 (10.9–12.4)	11.3 (10.7–11.7)	n.s
Phosphate (mg/dL)	4.4 (3.8–5.3)	4.6 (3.9–5.4)	n.s
CRP (mg/L)	0.7 (0.4–1.9)	0.8 (0.2–1.2)	n.s
25 OH vitamin D (ng/mL)	17.4 (11.3–27.1)	17.3 (13.6–25.8)	n.s
Cholesterol (mg/dL)	152 (123–159)	134 (106.4–153)	n.s
Height (cm)	163.5 (155.4–168.0)	160 (152.4–168.2)	n.s
Weight (kg)	69.0 (62.6–77.3)	60.0 (54.6–65.0)	*p* < 0.01
BMI (kg/m^2^)	25.9 (23.7–28.6)	23.4 (21.4–26.1)	*p* = 0.01
Brachial perimeter (cm)	26.9 (25.0–28.4)	25.4 (23.4–27.3)	n.s
Waist perimeter (cm)	97.7 (89.5–103.2)	89.3 (80.8–95.5)	*p* < 0.01
Hip circumference (cm)	99.7 (96.9–106.4)	98.8 (95.4–106.6)	n.s
Waist hip index	0.9 (0.9–1.0)	0.9 (0.8–0.9)	*p* < 0.01
Tricipital fold (cm)	1.1 (1.0–1.4)	1.2 (0.7–1.4)	n.s
Subscapular fold (cm)	14.5 (11.0–21.4)	12 (9.5–16.8)	n.s
Abdominal fold (cm)	15 (11.5–23.7)	20 (16–22.0)	n.s
Muscle mass (kg)	20.2 (17.2–23.3)	17.8 (15.7–20.0)	*p* = 0.02
Fat mass (kg)	24.3 (20.7–28.5)	20.4 (16.3–24.0)	n.s
FFM (kg)	48.4 (38.7–52.5)	40.2 (37.4–46.4)	*p* = 0.03
TBW (L)	35.5 (28.4–38.4)	30.4 (27.5–34.2)	*p* = 0.03
ECW (L)	16.4 (13.6–18.3)	15 (13–16.5)	n.s
LCW (L)	18.3 (15.1–20.3)	15.8 (13.8–17.8)	*p* = 0.02
ECW/ICW	0.9 (0.9–0.9)	0.9 (0.9–1.0)	n.s
BCM (kg)	23.9 (20.5–27.7)	21.3 (18.5–23.8)	*p* = 0.02
ECM (kg)	21.5 (17.1–22.9)	17.7 (15.5–21.5)	*p* = 0.05
FFMH (%)	73.5 (72.8–74.5)	73.5 (72.9–74.7)	n.s
Fluid excess (L)	0.6 (0.1–1.9)	0.4 (0.0–2.0)	n.s
HG criteria, n (%)	34% 11/32	14% 4/28	n.s
ASM criteria, n (%)	69% 22/32	50% 14/28	n.s
GS criteria, n (%)	34% 11/32	25% 7/28	n.s

MIS: malnutrition (extreme malnutrition (>10 pts.); very severe malnutrition (>7–10 pts.); and moderate–severe malnutrition (>5–7 pts.)); normonutrition (mild–moderate malnutrition (>2–5 pts.) or, if ≤2 points, normonutrition). FFM: fat-free mass, TBW: total body water, ECW: extracellular water, ICW: intracellular water, BCM: total cell mass, ECM: total extracellular mass. FFMH: fat-free mass hydration. HG: grip strength by dynamometry, ASM: appendicular skeletal muscle mass and GS: gait speed. n.s: no significant.

**Table 3:** Correlation matrix between sarcopenia diagnosis, nutritional and anthropometric parameters and MIS scale.

		HG (Probability)	HG + ASM (Confirmation)	HG + ASM + GS (Severity)
*Demographic data*				
Age (years)	r p	−0.2 n.s	−0.3 0.017	−0.3 0.018
*Anthropometric data*				
Albumin (g/dL)	r p	0.08 n.s	0.14 n.s	0.15 n.s
Total protein (g/dL)	r p	−0.04 n.s	0.07 n.s	0.03 n.s
Total cholesterol (mg/dL)	r p	0.04 n.s	0.29 0.03	0.33 0.01
Phosphate (mg/dL)	r p	0.045 n.s	0.33 0.01	0.27 0.04
*Analytical data*				
BMI (kg/m^2^)	r p	0.2 n.s	0.18 n.s	0.14 n.s
Weight (kg)	r p	0.09 n.s	0.33 0.01	0.21 n.s
Brachial Perimeter (cm)	r p	0.21 n.s	0.18 n.s	0.14 n.s
Abdominal fold (cm)	r p	0.18 n.s	0.3 n.s	0.23 n.s
Subscapular fold (cm)	r p	0.07 n.s	0.29 0.03	0.19 n.s
*Body composition data*				
Fat mass (%)	r p	0.02 n.s	−0.02 n.s	0.05 n.s
Fat-Free Mass (kg)	r p	0.19 n.s	0.32 0.01	0.17 n.s
Total Body Water (L)	r p	0.19 n.s	0.30 0.02	0.15 n.s
Extracellular Water (L)	r p	0.18 n.s	0.30 0.02	0.11 n.s
Intracellular Water (L)	r p	0.19 n.s	0.33 0.01	0.18 n.s
Body Cell Mass (kg)	r p	0.24 n.s	0.44 >0.001	0.30 0.02
Extracellular Mass (kg)	r p	0.1 n.s	0.25 0.05	0.1 n.s
Hydration Fat-Free Mass (%)	r p	0.12 n.s	−0.11 n.s	−0.13 n.s
Muscle mass (kg)	r p	0.24 n.s	0.45 >0.001	0.31 0.016
*Nutrition scale*				
MIS	r p	−0.14 n.s	−0.06 n.s	0.05 n.s

HG: grip strength by dynamometry, ASM: appendicular skeletal muscle mass and GS: gait speed. MIS: malnutrition–inflammation score. n.s: no significant.

## Data Availability

Databases are available upon request to the corresponding author.
